# The Effects of High-Pressure Processing on pH, Thiobarbituric Acid Value, Color and Texture Properties of Frozen and Unfrozen Beef Mince

**DOI:** 10.3390/molecules27133974

**Published:** 2022-06-21

**Authors:** Tuğba Şayin Sert, Fatma Coşkun

**Affiliations:** 1 Medical Faculty Catering, Trakya University, Balkan Campus, 22030 Edirne, Turkey; tunasert@gmail.com; 2 Food Engineering Department, Faculty of Agriculture, Tekirdağ Namık Kemal University, 59030 Tekirdağ, Turkey

**Keywords:** high pressure processing, beef meat, thiobarbituric acid, freezing, beef mince

## Abstract

In this study, beef mince (approximately 4% fat longissmus costarum muscle of approximately 2-year-old Holstein cattle) was used as a material. High-pressure processing (HPP) was applied to frozen and unfrozen, vacuum-packed minced meat samples. The pH and thiobarbituric acid (TBA) values of the samples were examined during 45 days of storage. Color values (L*, a* and b*) and texture properties were examined during 30 days of storage. After freezing and HPP (350 MPa, 10 min, 10 °C), the pH value of minced meat increased (*p* > 0.05) and its TBA value decreased (*p* < 0.05). The increase in pH may be due to increased ionization during HPP. Some meat peptides, which are considered antioxidant compounds, increased the oxidative stability of meat, so a decrease in TBA may have been observed after freezing and HPP. While the color change in unpressurized samples was a maximum of 3.28 units during storage, in the pressurized sample, it exceeded the limit of 10 units on the first day of storage and exceeded the limit of 10 units on the third day of storage in the frozen and pressurized sample. Freezing and HPP caused the color of beef mince to be retained longer. The hardness, gumminess, chewability, adherence, elasticity, flexibility values of the pressurized and pressurized after freezing samples were higher than those of the unpressurized samples during storage. On the other hand, the opposite was the case for the adhesiveness values. In industrial applications, meat must be pressurized after being vacuum packed. If HPP is applied to frozen beef mince, some of its properties such as TBA, color, and texture can be preserved for a longer period of time without extreme change.

## 1. Introduction

High-pressure processing (HPP) is a food-processing technology that does not require heat treatment, which the meat industry can use to produce fresh, safe, nutritious, high-quality, ready-to-eat, natural, and minimally processed meat products [1]. HPP causes less deterioration in the overall quality of processed foods compared to heat-treated foods [2]. However, administration of HPP under certain processing conditions (temperature (T), pressure (P), and time (*t*)) may affect the labile nature of proteins, particularly those found in (unprocessed) fresh meats [1]. Proteins can contain covalent bonds, disulfide bonds, hydrogen bonds and other interactions in their structures. Although HPP application cannot break covalent bonds, it can affect hydrogen bonds and other interactions. Oligomeric proteins can be dissociated in low-pressure applications (<150–200 MPa). However, significant oligomer changes are observed above 200 MPa. HPP applied above 200 MPa can affect the dissociation of proteins, and it can also provide recombination of dissociated oligomers. However, reversible unfolding of proteins can also be observed at 400–800 MPa [3]. Changes in the secondary structures of proteins occur only at very high pressures, as a result of the breaking of hydrogen bonds that provide the formation of the helical structure. This change in the secondary structure is usually irreversible [4]. High-pressure application causes volumetric reduction. It has been reported that a volume change of 500 mL/mol occurs in the dissociation of the lactate dehydrogenase enzyme [3]. With the effect of HPP, there is a decrease in molecular gaps and an increase in internal interactions [4]. With the increase in ionization in aqueous systems, changes occur in pH and a decrease in volume occurs as a result of the reactions [5]. In studies on myofibrillar proteins, one of the most important proteins in meat, it has been determined that HPP increases the solubility of these proteins and causes structural changes [6]. HPP application has a weak effect on connective tissue compared to myofibrillar proteins. Therefore, the connective tissue may limit the tenderness in meat after HPP application [7]. The effect of HPP application on the tenderness of meat occurs as a result of increasing the activity of proteolytic enzymes as well as physical strength [8]. Depending on the conditions applied, the appearance of the meat and other quality characteristics may be moderately to severely adversely affected [1]. Lipids may also be affected by HPP administration. Oxidation is one of the most important factors in the non-microbial degradation of meat [9]. HPP application causes myoglobin and oxymyoglobin in reduced state to convert to ferric form. Thus, fat oxidation is catalyzed by the effect of iron [4]. However, Orlien et al. [10] reported that the reason for the lipid oxidation of HPP application was not due to the catalytic effect of metmyoglobin; that is, the effect of the iron ion released by the pressure effect, but to the damage of the membrane. Orlien et al. [10] stated that pressure applications of 500 MPa and below for 10 min on chicken breast meat do not cause rancidity and 500 MPa is a critical value in this sense. Cheah and Ledward [11] determined that the pressure applied to pork minced meat at room temperature above 300 MPa significantly catalyzes lipid oxidation. The reaction products can easily react with proteins, leading to organoleptic modifications and loss of nutritional value [9].

Most of the HPP-treated meat products available on the market are either fermented, cured, or dried before HPP. HPP for raw meat is not considered a commercial application as it causes undesirable color changes [12,13,14], texture [15,16], and fat oxidation [17,18]. During application of high pressure, adiabatic heating occurs, which is equivalent to 3 °C per 100 MPa (in water). A change in meat color is observed as a result of HPP application. HPP, when applied at 10 °C, causes slight to obvious changes in visual appearance as pressure increases at pressures above 100 MPa. Therefore, HPP limits the commercial applications of fresh foods and is considered suitable for ready-to-eat foods [19]. Since HPP is effective on proteins, it can cause significant changes in color by affecting myoglobin in protein structure [4]. Cheah and Ledward [11] reported that 400 MPa causes irreversible denaturation of myoglobin in minced pork. Carlez et al. [12] applied HPP to the vacuum-packed beef minced meat at 10 °C for 10 min and determined that the L* color values increased significantly in the range of 200–300 MPa, and the color turned pink. They determined that the a* values decreased in the range of 400–500 MPa and the color turned into a gray-brown color. They found that while the oxymyoglobin ratio decreased in the range of 400–500 MPa, metmyoglobin increased. In addition, these researchers state that the denaturation of metmyoglobin under pressure is similar to the denaturation of heat, acid, and urea, and this is partly due to the breaking of hydrophobic interactions. In the study of McArdle [18], samples pressurized at 200, 300, and 400 MPa at 40 °C resulted in higher L* values, while b* values were lower when treated at 20 °C. The higher L* values observed were associated with higher protein denaturation at higher pressurization temperatures. HPP can be applied at ambient temperature or low temperature, or at high temperatures, with differential effects on meat proteins and texture [19]. It has been reported that freezing of meat may prevent discoloration in HPP at low temperatures [20]. 

Although non-thermal HPP (typically 400–600 MPa) effectively reduces a range of pathogens, it is not sufficient to inactivate bacterial spores and mold as some can withstand >1000 MPa [21,22,23]. In addition, improper processing or storage conditions can cause microbial retention, which can lead to deterioration of quality. Therefore, the addition of conventional physical barriers such as temperatures >50 °C or <20 °C to 30 °C or low pH values can be used to increase the effectiveness of the HPP [23,24]. 

There are very few studies on freezing and high-pressure–low-temperature-combined operation. These studies were performed by Fernández et al. [14] (650 MPa, −35 °C, 10 min) in beef, Realini et al. [25] (400 and 600 MPa, −15 and −35 °C, 6 min.) in pork fillet, and Vaudagna et al. [26] (400 and 650 MPa, −30 °C, 1 and 5 min) in dried beef carpaccio. In these studies, it was stated that the combined applications of freezing and low temperature preserved the texture better. These findings were associated with freezing to reduce pressure-induced muscle fiber elongation.

Few studies have been performed on the combination of freezing and high-pressure treatment in beef. Since pH, thiobarbituric acid, color, and texture properties are the leading quality criteria of meat, the effect of this application on the quality parameters may determine the usability of the application. In this study, the effects of high-pressure processing on pH, thiobarbituric acid, color, and texture properties of frozen and unfrozen beef mince during storage was investigated.

## 2. Results and Discussion

### 2.1. pH

Right after HPP, the pH values of the pressurized samples were about 0.07–0.08 units more than the pH values of the unpressurized samples (Table 1). An increase of 0.1–0.2 units in the initial pH of raw beef was reported with the loss of acid groups in meat by protein denaturation after pressurization [15,18]. In Mc Ardle’s study, high-pressure processing at 200 MPa did not change the pH values of the beef, regardless of the pressurization temperature. Conversely, increases in pH values (pb0.01) were detected at higher pressure levels (300 and 400 MPa) [18].

While the pH values of the pressurized samples increased until the 15th day of storage and then decreased, the pH values of the unpressurized samples decreased until the 15th day of storage and then increased. In the study of De Alba et al. [27], the pH (450 MPa, 5 min) of pressurized beef carpaccio increased by 0.01 units after 30 days of storage at 8 °C. The pH of unpressurized sample decreased by 0.81 units until the 15th day of storage. Then, it increased by 0.09 units until the 30th day of storage.

In this study, pressurization and freezing caused the pH to rise and remain high during storage. The pH of a frozen food can change with an increase in the concentration of solutes (with the formation of ice) and precipitation of a salt at a supersaturated level. During freezing of food, a change in salt concentration and composition can cause a pH change that can contribute to changes in the reactivity of proteins [28] and enzyme activity (production of acidic or alkaline substances or the buffering capacity of the product). Storage temperature is also important [29]. The increase in pH after HPP has been attributed to the reduction of acidic groups present in meat as a result of conformational changes associated with protein denaturation [30]. Clarke, Means, and Schrnidt [31] found no difference in pH of ground beef due to changes in salt level; However, Poulanne and Terrell [32] found that formulating sausages with 4% salt increased their pH compared to those formulated with 2%. Macfarlane, McKenzie, and Turner [33] found that applying 150 MPa pressure to bovine muscle significantly increases pH values due to loss of free protons due to the redistribution of ions caused by increased ionization at high temperatures [34]. A study by Van den Berg [29] found that the pH of beef rose from 5.6 to 5.9 during freezing at −10 °C. 

### 2.2. TBA (Thiobarbituric Acid) 

Lipid oxidation is one of the main factors affecting processed meat quality [35]. The composition of fatty acids is one of the most important components that can change during processing, affecting sensory perception of food and the nutritional value of the meat [36]. TBA values were used in the present study as a measure of lipid oxidation. TBA values of all samples were found to be higher than expected for the start of storage (Table 1). This can be attributed to the fact that meat was passed through a mincing machine two times before becoming ground before HPP, and then it was mixed by a food robot for 10 min. Mechanical processing of meat before high pressure (slicing or mincing) creates a strong prooxidant effect [37,38]. The amounts of TBA were found to be lower in F (frozen) sample and F + P (frozen + pressurized) sample than in C (unfrozen and unpressurized) and P (pressurized) samples on day 1 of storage (*p* < 0.05). According to these results, pressure-induced oxidation did not occur immediately after HPP. Studies showed that the oxidation did not increase immediately after the HPP; rather, it increased during storage [37,39]. 

On the third day of storage, the amount of TBA increased in the F + P sample (*p* < 0.05), while in all other samples, it decreased. Because the samples were vacuum packed, it was thought that the oxygen level decreased and the TBA level decreased. Contrary to the F + P sample, the decrease in the TBA value of the P sample on the third day of storage suggested that HPP did not cause the increase in the TBA value of the F + P sample. As a result of freezing, ion concentrations increase, which leads to oxidative reactions, dehydration of cell membranes, and exposure to oxidation of membrane phospholipids. An increase in inorganic ions can exhibit specific effects. They influence the activity of muscle enzymes and establish cross-links between nearby peptide chains. These ions, while helping to form lipid–protein complexes, can accelerate lipid oxidation [40]. The decreases in TBA were attributed to several factors. Some meat peptides are considered antioxidant compounds, thus increasing the oxidative stability of meat [41]. In fact, one study concluded that meat stability was positively affected by the content of antioxidant peptides [42]. The mechanism of action of peptides as antioxidants is not fully understood, but it is clear that they scavenge radicals, reduce hydroperoxides, and chelate metals. The antioxidant activity of peptides largely depends on the amino acids they contain in their chemical structure [43]. On the 15th day of storage, the TBA values of all samples increased (C sample had the highest TBA value) (*p* < 0.05). The TBA values of all samples decreased from the 30th day of storage to the end of storage. Malonaldehyde, which is formed as a result of oxidation of oils, is highly oxidized and turns into alcohol and acids, and the decrease in malonaldehyde concentration decreases TBA values [44]. 

Witte et al. [45] reported that the TBA value for consumable meat products was 1–2 mg MA/kg. In this study, since beef is a meat type that is not susceptible to oxidation in terms of unsaturated fatty acids, even on day 15 of storage, when TBA values were highest, all meat samples may have been found to be consumable in terms of TBA. At the end of storage, the TBA values of all samples were found to be lower than the TBA values at the beginning of storage (*p* < 0.05). The highest TBA level at the end of storage was seen in the C sample. Zhu et al. [46] and Andrés et al. [47] reported that raw material quality, time between animal cutting and meat processing and product variety may have different effects on lipid oxidation development after HPP. Cheah and Ledward [17] reported an increase in the amount of TBE in minced pork during the storage after application of high pressure at 200–800 MPa (20 min, 20 °C). They have stated that when HPP is applied below 300 MPa there is little effect on lipid oxidation, but when applied at higher pressures, it may have a significant effect. Therefore, the 350 MPa (10 min, 10 °C) HPP parameter used in this study is not a pressure value large enough for oxidation formation in the minced beef. The reason for the low TBA value of the F sample at the end of storage is that this sample stored at −21 °C. TBA values of pressurized samples (P, F + P) were also low at the end of the storage. Accordingly, it can be said that freezing and pressing reduces TBA values. 

### 2.3. Color

Unpressurized samples (C and F) were accepted as control samples. At the beginning of storage (Day 0), only these samples were analyzed. The first of the color analyses was performed 18 h after packaging, on sample C stored at 4.0 ± 2 °C and sample F stored at −21.0 ± 2 °C. The F sample was thawed before analysis. Freezing at −21.0 ± 2 °C and then thawing did not have a significant effect on the color of minced beef. The values were shown in the Day 0 column of Table 2. All samples were analyzed from the first day of storage.

The type of meat, pressure level and temperature, chemical state of myoglobin, and curing are important in the effect of pressurization on meat color [1]. Although there is very slight color change at pressure levels below 200 MPa, application of pressure above 200 MPa makes meat appear much paler than unpressurized meat due to protein denaturation and coagulation. Structural modifications lead to changes in the proportions of absorbed, refracted, and reflected light. This causes increased light-scattering. Thus, the meat has a paler appearance [1,48]. HPP also affects the redox chemistry of myoglobin (Mb). Studies have shown that the ferrous Mb form disappears within the first day (300 to 800 MPa) after HPP administration [1,49].

L* values of samples were shown in Table 2. The HPP caused an increase in the L* values of the samples (*p* < 0.05). Increases in L* values immediately after HPP were reported in similar studies [12,13]. During storage, L* values of the pressurized samples were higher than those of the unpressurized samples (*p* < 0.05). The L* value of the C sample was not significantly changed during storage (*p* > 0.05). These results are in agreement with the results of the study conducted by Çiçek et al. [50]. The non-change of L* values may be due to vacuum packaging. During storage, the L* value of the P sample increased by about two units, while the L* value of the F + P did not change (*p* > 0.05). On the 15th and 30th days of storage, the L* values of the P sample were found to be higher than the F + P sample (*p* < 0.05). According to these results, the color of beef mince samples could be preserved for a longer time when freezing and HPP were performed. De Alba et al. [51] also found that L* values of pressurized beef carpaccio samples were higher than in untreated samples (*p* < 0.05). Additionally, they reported that L* values tend to increase during 15 days of storage after HPP. No significant change in L* values was observed until the 30th day of storage. 

McArdle et al. [52] reported that L* values of pressurized (35, 45, 55 °C, 400, 600 MPa) beef samples remained stable during storage (for 30 days at +4 °C) and indicated that L* values of the control samples were lower (*p* < 0.001) than those of the pressurized samples during the storage. The lighter appearance was attributed to globin denaturation and/or heme group displacement or release by Carlez et al. [12].

a* values of samples were shown in Table 2. a* (redness) decreased after HPP (*p* < 0.05), and redness of the F + P samples was found to be higher than the P samples in all days of storage. Freezing protects beef meat against the detrimental effect of pressure on color as the fresh beef meat normal color was recovered after thawing in frozen beef pressurized at sub-zero temperature; the effect was probably milder and reversible. Thus, on thawing, myoglobin might recover its native conformation, and consequently, fresh meat’s normal color could be recovered [51]. Freezing before HPP causes preservation of meat redness during storage. This may be due to the fact that the molecular transport is reduced in the frozen and pressurized samples, and the inhibition of the movement of protein molecules which give the meat color. In addition, water is important in protein expansion, and protein groups are more stable in a dry state [53]. Redness was reduced in the C sample on the third day of storage and decreased in the P sample on the 30th day (*p* < 0.05). In F + P sample, redness (a*) did not change during storage (*p* >0.05). The a* values of the pressurized samples during storage were lower than those of the unpressurized samples (*p* < 0.05). Kim et al. [54] found that the redness of the pressurized beef meets at 500 and 600 MPa for 5 min at 15 °C was lower than the control sample. Bajovic et al. [55] and Bak et al. [49] reported significantly less redness with 300 MPa HPP. The reduction in a* values due to HPP has been attributed to the myoglobin content reduced and the formation of met myoglobin by researchers [12]. De Alba et al. [27] found that the a* values of pressurized beef carpaccio samples lower than a* values of control samples on the first day of storage. In their study, the a* values of all samples decreased during storage (at 8 °C). 

b* values of samples were shown in Table 2. While the b* values of the pressurized samples did not change during storage (*p* > 0.05), the b* values of the unpressurized samples increased on the seventh day of storage (*p* < 0.05), then showed a decreasing trend again. Carlez et al. [12] found no significant difference between the b* values of the pressurized and unpressurized fresh beef mince samples. There was no significant change in b* values during 14 days of storage. In this study, b* values of pressurized samples were similar to those of unpressurized samples (*p* > 0.05). The b* values of the F + P samples were found to be higher on all days of storage (except the 15th day) compared to the other samples. Variations in yellowness of meat products have been related to changes in the chemical state of myoglobin [56]. Although statistically insignificant, the b* values of F + P samples decreased over 30 days of storage. De Alba et al. [51] determined that b* values of sliced dry-cured decreased (despite the increase in some days of storage) at the end of storage (60 day at 8 °C) after HPP (400, 500, 600 MPa, 5 min). In a study conducted by Bulut [57], frozen and unfrozen beef mince were exposed to HPP (300 MPa, for 5 min, at 10 °C). The L* value of the frozen and unfrozen samples increased at the end of the pressurization. The a* values of unfrozen samples did not change after pressurization, but the a* values of frozen samples decreased after pressurization. b* values did not change in both applications. The highest value of *ΔE* was detected in the frozen pressurized sample and the lowest value was detected in the frozen control sample.

Lowder et al. [58] reported that no color change was observed in raw bovine meat frozen at −30 °C and pressurized at 550 MPa for 4 min. Decreases in the bleaching of red meat color as a result of freezing application before a HPP treatment were shown by Fernández et al. [14] for bovine meat and by Realini et al. [25] for pig carpaccio.

The total color difference *ΔE* take into account the evolution of the three color parameters (a*, b* and L*). The *ΔE* values of the F sample and the C sample on day 1 of storage were 1.04 and 0.00, respectively. The *ΔE* values of the F + P sample and the P sample on day 1 of storage were 7.6 and 10.4, respectively (Table 2). The mean 10 unit change in *ΔE* value is reported as a remarkable change in terms of color change in the literature [13]. In this study, the *ΔE* value of the P sample reached the limit of 10 units on the first day of storage, whereas the *ΔE* value of F + P sample reached the limit of 10 units on the third day of storage. The *ΔE* value of the C sample was 0.00 on all analyzed days of storage. The *ΔE* value of the F sample increased up to 3.28 on the seventh day of storage and then decreased. Freezing of the samples before HPP resulted in better protection of the color at the beginning of storage, but the preservation effect did not continue during storage. Fernández et al. [14] found that the color values of pressurized beef samples did not change during 45 days at −18 °C storage. Researchers have reported irreversible protection in the color of beef meet frozen and pressurized at low temperature. In that study, the color values after 45 days were below the limits. According to Cheftel and Culioli [59], high pressure causes dramatic changes in the color of fresh meat, and thus makes the commercialization of HPP fresh meats difficult, since they lack the typical color of fresh meat from the consumer’s perspective. However, these changes are not relevant if the products are further processed, such as, for example, into hamburger patties for food service [55].

### 2.4. Texture

Hardness, gumminess, adhesiveness, chewability, adherence, elasticity, and flexibility values of samples during storage are shown in Table 3.

The hardness was determined to be higher in the pressurized samples during storage compared to the unpressurized samples (*p* < 0.05). It has been reported that HPP can cause softening or hardening in meat protein depending on temperature, pressure, and time, and this is caused by protein denaturation, aggregation, or gelation. Therefore, it has been suggested that processing conditions should be carefully controlled to increase the tenderizing effect in meat muscle [59,60]. The hardness value of the F + P sample decreased during the shelf life. Regarding this, Zare [61] stated that the decrease in tissue hardness of pressurized samples during storage may be due to incomplete inactivation or reactivation of protease enzyme activities during storage. Angsupanich and Ledward’s study [62] showed a decrease in acid, neutral and alkaline proteases, and denaturation of actin and sarcoplasmic proteins at 200–400 Mpa. This leads to the increases in gumminess, hardness, and adhesiveness. Above 400 Mpa, this effect diminished. The hardness of the F + P sample was higher than the hardness of the P sample before the 30th day of storage (*p* < 0.05). The increases in the hardness of the pressurized samples during storage may have resulted from protease inactivation in meat. Another possibility was that the water retained in the myofibrils after HPP was excreted during storage. While the hardness value did not change during storage in the F sample (*p* > 0.05) the hardness decreased in the C sample from the third day of storage (*p* < 0.05). It can be said that as a result of the increase in the level of microorganisms and the activity of enzymes, the myofibrils in the meat begin to break down, and a loss of hardness may have occurred, starting from the surface of the meat.

The gumminess increased in the pressurized samples compared to the unpressurized samples and was higher than the unpressurized samples during storage (*p* < 0.05). The highest gumminess value of the P sample was determined on the seventh day of storage. The highest gumminess value of the F + P sample was determined on the first day of storage (which is the highest value among all samples and between all days) and decreased during storage. While the gumminess had the lowest value in the C sample on the third day, it increased after the third day. In their study on chicken breast fillets, Kruk et al. [63] determined that cohesiveness, gumminess, hardness, and chewiness increased with the effect of high pressure. The 450 and 600 MPa pressure inflicted the most detrimental effects.

During storage, the chewability of the pressurized samples was higher than the unpressurized samples, and the chewability of the F + P sample was higher than that of the P sample (*p* < 0.05). While the chewability of the pressurized samples decreased after the seventh day (*p* < 0.05), a decrease was observed in the C sample after the first day (*p* < 0.05). Hardness, springiness, cohesiveness, and chewiness reflect the degree of muscle softness, the ability to resist external force recovery, the tightness of muscle tissue bonding, and “bite strength” [60]. Cheftel and Culioli [59] and Torres and Velazquez [60] stated that the chewability of meat increased with the application of HPP; however, HPP had little effect on the strength of the connective tissue, and the chewability values were proportional to the strength of the internal bonds. The effect of HHP on textural properties can be explained by myofibrillar protein denaturation and gel formation [64]. In the study by Ma et al. [65], chewability increased with the application of HPP, and the increase was greater at pressures higher than 200 MPa. Similarly, in this study, the chewability values increased with pressure. However, the decrease in chewability in the F + P, P, C samples during the storage may be due to weakening of the internal bonds as a result of biochemical and physicochemical changes in meat.

During storage, the adhesiveness was determined to be lower in the pressurized samples compared to the unpressurized samples. On the first day of storage, the adhesiveness level of the P sample was lower than in the other samples (*p* < 0.05). There was a significant decrease in the adhesiveness values of the pressurized samples on the 30th day (*p* < 0.05). The reduction in firmness, stickiness, and chewiness under temperature and pressure was attributed to increased denaturation of myosin and collagen by Angsupanich and Ledward [62].

Flexibility was higher in pressurized samples compared to unpressurized samples during storage (*p* < 0.05). Flexibility increased with pressure application and flexibility values of P and P+F samples were found to be similar during storage (*p* > 0.05). Elasticity decreased on the 15th and 30th days of storage in the pressurized samples. The increase in flexibility resulting from the increase in the internal bonding level of meat samples with pressurization is an expected situation, and it has also been detected by researchers [59,60].

There was no significant change in elasticity and adherence (internal stickiness) values during storage in all samples (*p* > 0.05). Elasticity was determined higher in pressurized samples compared to unpressurized samples (*p* < 0.05), and the adherence was similar (*p* > 0.05) on all days of storage. Although the effect of pressure on adherence was not significant, it increased. 

In traditional HPP applications, changes in the texture of the meats were detected. In a study, it was determined that the hardness of pork increased with the effect of moderately high pressure (215 MPa, 15 s, at 33 °C) [66]. It has been reported in the literature that consumers prefer beef that has been treated with HPP up to 200 MPa [67]. However, while an increase in microbial quality was achieved in HPP applications above 300 MPa in general, it was reported that the color, texture, and taste of meat were adversely affected at these pressures. The effect of applying HPP to meat depends on pressure, temperature, time, muscle, and postmortem time, and it has been reported that meat can be hardened as well as tenderized [20]. Meat tenderness (tenderization) depends on the durability of myofibrillar proteins and the presence of connective tissue and other stromal proteins [16]. For this reason, this technology must be optimized in accordance with the purpose in order for a successful commercial application to take place.

Since the weak bonds that stabilize the secondary, tertiary, and quaternary structures of proteins respond differently to heat and pressure applications, high-pressure treatment at different temperatures creates different effects on meat texture [68]. Denaturation of proteins occurs due to the destabilization of non-covalent interactions in the tertiary structure. As a result of HPP, a small amount of unfolding occurs, exposing the hydrophobic regions of the processed protein. This is accepted as the reason for the aggregation (clustering) of proteins [16]. During the denaturation process induced by HPP, muscle proteins can dissolve or precipitate depending on the pressure used. Changes in the 100–300 MPa range are normally reversible, but when the application pressure is higher than 300 MPa, the changes that occur are usually irreversible [69]. Hydrophobic interactions, the main forces stabilizing the quaternary structure, are very sensitive to pressure. According to Okamoto et al. [70], on the other hand, in the basic mechanism of tissue change with HPP, different types of meat undergo tissue change due to a decrease in protein volume under pressure. After HPP, the volume of the protein decreases due to the compression of the internal cavities. Pressure-dependent gelation of meat proteins depends on the protein system and HPP processing conditions (pressure level, time, and pressurization temperature, etc.) [71]. It is generally accepted that protein oxidation could lead to undesirable texture changes, including tenderness [72,73] and water-retention properties [74,75], in fresh meat and processed muscle foods [76].

In a study conducted by Fernández-Martin et al. [77], pork and beef muscles were subjected to 200 MPa and −20 °C with or without water freezing. Protein denaturation was greater when freezing occurred. Connective proteins remained practically unaltered by pressurization and/or freezing. Structural changes in the muscle at sarcomere levels caused by pressurization were more severe when freezing occurred.

## 3. Materials and Methods

### 3.1. Materials

In this study, the longissmus costarum muscle of approximately 2-year-old Holstein cattle (mean 4% fat) was used. Meat pieces taken from carcasses were placed in polyethylene boxes and brought to the laboratory under cold conditions. Beef mince was obtained by passing the meat through a refrigerated meat grinder twice, using a grinder plate with a 3 mm hole. The samples were refrigerated at 4.0 ± 2 °C before being prepared for analysis or pressure processing.

### 3.2. Preparation of Beef Mince Samples

The beef was homogenized for 5 min using a hand mixer (Bosch, Stuttgart, Germany). Approximately 10–12 g samples were packed in sterile bags (approximately 3 × 10 cm in size obtained by heat-sealing from stomacher bags) and then packed using a vacuum-packaging machine (MV-20, Lipovak, Gebze, Turkey). The samples were then vacuumed in small plastic bags made of Polyamide/ Polyethylene (oxygen permeability 10.4 cc/100 in^2^/day; moisture permeability 0.55 g/100 in^2^/day; weight 83.7 g/m^2^ and thickness 90 μ). repackaged. 

### 3.3. Storage in Deep-Freezer and Refrigerator before HPP

The control sample (C) was stored at +4 °C from the beginning until the end of storage, and the frozen sample (F) was stored at −21 °C from the beginning until the end of storage. The pressurized sample (P) was the sample that was pressurized after being stored at +4 °C from the beginning of the storage to the first day. That sample was stored at +4 °C until the end of storage after pressurization. The frozen and pressurized sample (F + P) was the sample that was thawed and then pressurized after being stored at −21 °C from the beginning of the storage to the first day. That sample was not refrozen after pressurization, but stored at +4 °C. As the deep freezer, an INDESIT (UIAA 10 TK) brand deep freezer, annual energy consumption (256 kWh/year), a 194 lt volume, home-type deep freezer with a static cooling system was used. The thawing process was also carried out in a home-type refrigerator (VESTEL BZP-XL4303WY No-Frost Combi Refrigerator)

### 3.4. High-Pressure Treatment

A high-pressure system (model MSE-CIP-WB-5500, MSE Teknoloji Ltd., Gebze, Turkey) with a working volume of 0.7 L was used for the HPP. Details of the HPP system were given by Bulut [57]. A mixture of Propyleneglycol (1,2-propanediol; Kimetsan Co., Ltd., Ankara, Turkey), and water at a ratio of 55/45% (*v*/*v*) was used as the pressure-transmitting medium. The temperature of the pressurized vessel and the temperature of the pressure-conducting medium within the chamber were controlled by means of a circulating cooler (model RE1050S, Lauda Dr. R. Wobser GmbH and Co. KG., Lauda-Königshofen Germany). The pressure was increased to a test pressure (350 MPa) at a rate of about 8–10 MPa/s. Pressurization continued for 10 min at 10 °C. The pressure was released manually within approximately 20–30 s by gradually opening the pressure relief valve after the required time had elapsed. A K-type thermocouple mounted in the center of the top cover of the pressure chamber was placed close to the sample to monitor the temperature of the chamber during the pressure treatments. Temperature and pressure data were recorded by the software controlling the HPP system.

### 3.5. Storage in Deep-Freezer and Refrigerator after HPP 

The F sample (frozen) was stored at −21.0 ± 2 °C, F + P (frozen before pressurization and pressurized). The C (control and unfrozen) and P (unfrozen before pressurization and pressurized) samples were stored at 4.0 ± 2 °C for 45 days. Temperatures during storage were monitored and recorded using a temperature data logger (Huato HE800, Shenzhen, China). The pH and TBA analyzes of the samples were performed on the 1st, 3rd, 7th, 15th, 30th, and 45th days of storage. Color analysis was performed at the beginning of the storage, on the 1st, 3rd, 7th, 15th, and 30th days.

### 3.6. pH Analysis

After adding 100 mL of purified water to the 10 gr of mince sample, the samples were homogenized at 200 rpm in Stomacher (400 sq. SEWARD) for 1 min. Using a pH meter (S220Metter-Toledo 8603 Switzerland) adjusted with buffer solution, the pH value was measured by the method described by Gökalp et al. [78].

### 3.7. TBA (Thiobarbituric Acid) Analysis

The oxidation levels of the fat of the samples were determined using a 2-thiobarbituric acid method. The analysis was carried out according to the method indicated by Tarladgis et al. [79]. Next, 10 g of the homogenized sample was transferred to a 1000 mL balloon. After the addition of 97.5 mL of purified water, 2.5 mL of 4 N HCl solution, boiling water and glycerol, the balloon was connected to the reflux cooler. The distillation process was carried out in a jacketed heater. The distillation was continued until 50 mL of distillate was accumulated. Then, 5 mL of distillate and 5 mL of TBA reagent (0.2883 g TBA, 100 mL of 90% glacial acetic acid) were added to the glass tubes and the covers of the tubes were closed. The blank sample was prepared by adding 5 mL of purified water and 5 mL of TBA reagent into a glass tube. The prepared tubes were kept in a boiling water bath for 35 min, then cooled and read on a spectrophotometer at a wavelength of 538 nm. The absorbance value obtained was multiplied by factor 7.8 and the amount of malonaldehyde present in 1000 g of the sample was determined in mg. The TBA values of the samples were evaluated according to the criteria of Varlık et al. [80]. TBA tests were carried out on day 1, 3, 7, 15, 30, and 45 on triplicate samples and the average counts were used for calculations.

TBA value (mg malonaldehyde/kg sample) = 7.8 × A.

A = Absorbance value at 538 nm.

### 3.8. Color Measurements

Color measurements were performed using a Konica Minolta colorimeter (model CM-5, Minolta Co., Ltd., Osaka, Japan). Measurements were carried out using a 0.3 cm aperture port, Illuminant D65, and 10° standard observers and were calibrated using CR-A44. Three parallel samples (10 g each) were prepared for each application. All samples were awaited at about 25 °C for 20 min, then placed in a Petri dish and mixed with a spatula for about 1 min until a homogenous mixture was obtained. The aim was to allow reoxygenation. Then, samples were placed into the instrument’s Petri dish for color measurements. Commission Internationale de l’Eclairage (CIE) lightness (L*), redness (a*) and yellowness (b*) values of beef mince were measured three times on each sample by the instrument, and the average value was recorded. The total color difference (*ΔE*) was determined as an estimate of color changes and was calculated as suggested by Jung et al. [13]. The investigations were carried out in comparison with untreated control samples and the color values of unpressurized samples were used to calculate *ΔE*. Color tests were carried out on day 1, 3, 7, 15, and 30 on triplicate samples and the average counts were used for calculations.

*ΔE* = [(L* − *L*_0_)^2^ + (a* − *a*_0_)^2^ + (b* − *b*_0_)^2^]^1/2^ (1)

### 3.9. Texture Analysis

The minced meat was placed on Petri plates (4 cm diameter, 1 cm height) in such a way that there was no air, and vacuum packaging was performed on the same day. Frozen (F) and unfrozen (C) samples were prepared by keeping half of the meat samples in the freezer at −21.0 ± 5 °C for 24 h and the other half at +4.0 ± 2 °C for 24 h. Unfrozen (C), frozen (F), unfrozen pressurized (P), and frozen pressurized (F + P) samples were reshaped for 1 min with a spatula before analysis, and then texture analysis was performed. Texture analysis was performed on the 1st, 3rd, 7th, 15th, and 30th days of storage.

For texture profile analysis, the measurement method proposed by Bourne [81] was applied using the TA HD PLUS Textured Analyzer (Stable Microsystems, Godalming, Surrey, UK). For this purpose, the test speed was chosen as 1.2 mm/s and it was compressed to 50% of its original height using a cylindrical probe with a diameter of 100 mm. Measurements were made with a trigger force of 5 g and a waiting time of 5 s between two compressions. Average values were calculated by making 3 measurements for each parameter. Hardness, gumminess, adhesiveness, chewability, adherence, elasticity, and flexibility parameters were evaluated.

### 3.10. Statistical Analysis

Analysis results were evaluated with variance analysis (one-way ANOVA). The significance level was determined as *p* < 0.05 in comparing the differences between processes (freezing and pressurization) and days. The importance of each variable was evaluated with the post hoc test. Levene test Tukey in the case of *p* > 0.05 Tamhane’s multiple comparison tests in the case of *p* < 0.05 were used. All data and standard errors deviations of data were evaluated using SPSS 16. All analyses and measurements were repeated in triplicates.

## 4. Conclusions

HPP may adversely affect the quality characteristics of raw meat depending on the application conditions and the type of meat. The pH value increased insignificantly with the pressurization and freezing processes and remained high during storage. Apart from the influence of HPP, conditions to which meat is exposed during preparation before HES (mincing and mixing during homogenization) may affect post-pressurization TBA values. Preparations should take a short time. In this study, vacuuming of meat and low fat content (average 4%) helped prevented the increase in TBA. Freezing and pressurization (350 MPa, 10 min, 10 °C) caused the TBA to decrease during storage and remain lower than the TBA of the control sample. It was thought that the increase in TBA on the 15th day of storage was not dependent on the HEPP but increased with storage. Freezing and pressurization provided longer preservation of meat color than pressurization without freezing. Extending the storage period reduces this protection. The L* value increased with pressurization. However, freezing reduced the L* increase. During storage, the hardness, gumminess, chewability, flexibility, and elasticity of the pressurized samples were higher than the unpressurized samples. The hardness gumminess and chewability of frozen and pressurized samples decreased during storage. Adhesiveness was found to be lower in pressurized samples compared to unpressurized samples during storage. No significant effects of pressure and freezing on adherence were observed. It has been reported in the literature that consumers prefer HPP-treated beef up to 200 MPa. However, in general, while an increase in microbial quality is achieved in HHB applications above 300 MPa, it has been reported that the color, texture, and flavor of the meat are adversely affected at these pressures. This technology needs to be optimized for this purpose. Freezing raw meat prior to HPP may allow a lower pressure level and exposure time. This can result in lower costs. The studies to be carried out may provide the opportunity to detect the application parameters and minimize the negative effects of the application.

## Figures and Tables

**Table 1:** pH, thiobarbituric acid values of control, frozen and pressurized (350 MPa, 10 min., 10 °C) after frozen and pressurized without frozen samples during the storage (F samples at −21 °C; other samples at +4 °C).

Analysis	Treatment	Day 1	Day 3	Day 7	Day 15	Day 30	Day 45
pH	C	5.89 ± 0.02 ^aA^	5.80 ± 0.10 ^bA^	5.43 ± 0.03 ^bC^	5.51 ± 0.04 ^aC^	5.56 ±0.01 ^aB^	5.64 ± 0.01 ^aB^
	F	5.90 ± 0.09 ^aB^	5.91 ± 0.05 ^aB^	5.99 ± 0.03 ^aB^	5.96 ± 0.02 ^bB^	6.00 ± 0.02 ^bB^	6.23 ± 0.06 ^bA^
	P	5.97 ± 0.02 ^aB^	6.01 ± 0.01 ^aB^	6.01 ± 0.02 ^aB^	6.05 ± 0.05 ^aA^	5.96 ±0.03 ^aB^	5.86 ± 0.01 ^aC^
	F + P	5.98 ± 0.03 ^aB^	6.02 ± 0.01 ^aB^	5.97 ± 0.01 ^aB^	6.05 ± 0.03 ^aA^	5.85 ± 0.03 ^aB^	5.73 ± 0.22 ^aC^
TBA mg MDA equivalent/kg	C	0.83 ± 0.10 ^aB^	0.73 ± 0.02 ^bB^	0.69 ± 0.01 ^bB^	2.01 ± 0.05 ^aA^	0.88 ± 0.03 ^aB^	0.70 ± 0.04 ^aB^
	F	0.62 ± 0.03 ^bB^	0.55 ± 0.05 ^cB^	0.57 ± 0.02 ^cB^	0.83 ± 0.01 ^cA^	0.66 ± 0.00 ^bAB^	0.38 ± 0.02 ^cC^
	P	0.80 ± 0.03 ^aB^	0.67 ± 0.02 ^bC^	0.67 ± 0.03 ^bC^	0.85 ± 0.03 ^cA^	0.66 ± 0.01 ^bC^	0.46 ± 0.03 ^bD^
	F + P	0.56 ± 0.02 ^bB^	0.91 ± 0.02 ^aA^	0.89 ± 0.01 ^aA^	1.01 ±0.02 ^bA^	0.59 ± 0.03 ^cB^	0.26 ± 0.03 ^dC^

C—unfrozen, unpressurized; F—frozen, unpressurized; P—unfrozen before HPP, pressurized; F + P—frozen before HPP, pressurized. Results and standard errors are mean values of three replicates. Different letters (a, b, c, …) in the same column indicate differences among values. Different letters (A, B, C, …) in the same row indicate differences among values (*p* < 0.05).

**Table 2:** CIE L*, a* and b* values of control, frozen and pressurized (350 MPa, 10 min, 10 °C) after frozen and pressurized without frozen samples during the storage (F+ samples at −21 °C; other samples at +4 °C).

Samples	Day 0			Day 1				Day 3				Day 7				Day 15				Day 30			
	L*	a*	b*	L*	a*	b*	*ΔE*	L*	a*	b*	*ΔE*	L*	a*	b*	*ΔE*	L*	a*	b*	*ΔE*	L*	a*	b*	*ΔE*
C	39.05± 0.15 ^b^	14.71± 0.08 ^a^	15.04± 0.25 ^b^	39.14± 0.17 ^bA^	14.68± 0.17 ^aA^	15,06± 0.25 ^bB^	0.00 ± 0.00	38.27± 1.06 ^bA^	12.18± 0.65 ^bB^	15.80± 0.42 ^abAB^	0.00 ± 0.00	40.01± 0.05 ^bA^	13.45± 0.06 ^bA^	16.43± 0.05 ^aA^	0.00 ± 0.00	39.34± 0.88 ^cA^	12.65± 0.57 ^aB^	15.72± 0.59 ^aAB^	0.00 ± 0.00	39.23± 0.59 ^cA^	12.23± 0.11 ^aB^	15.14± 0.70 ^bB^	0.00 ± 0.00
F	39.61± 0.10 ^a^	14.08± 0.50 ^b^	15.64± 0.07 ^a^	39.37± 0.42 ^bA^	14,05± 0.10 ^aAB^	15,72± 0.17 ^bAB^	1.04± 0.08 ^cB^	37.43± 0.55 ^baB^	13.53± 0.13 ^aB^	13.74± 0.16 ^bB^	2.84 ± 0.74^bA^	37.81± 0.21 ^cB^	15.68± 0.16 ^aA^	15.97± 0.97 ^aAB^	3.28± 0.12 ^cA^	37.76± 0.15 ^dB^	13.89± 0.14 ^aB^	13.98± 0.53 ^bB^	2.78± 0.32 ^bA^	37.05± 0.23 ^cB^	12.27± 0.28^aC^	14.03± 0.61 ^cB^	2.46 ± 0.45 ^cA^
P				47.68± 0.76 ^aB^	8.92 ± 0.27 ^cAB^	15.44± 0.60 ^bA^	10.4± 0.70 ^aA^	47.81± 0.27 ^aB^	8.52 ± 0.45 ^dAB^	15.32± 0.04 ^bAB^	10.24± 0.73 ^aA^	48.10± 0.15 ^aAB^	9.56 ± 0.61 ^dA^	15.37± 0.10 ^aAB^	9.05± 0.25 ^bB^	49.34± 0.12 ^aA^	8.03 ± 0.36 ^cAB^	15.41± 0.42 ^aA^	11.03± 0.79 ^aA^	49.36± 0.89 ^aA^	7.06 ± 0.69 ^cB^	14.82± 0.85 ^bB^	11.37± 1.36 ^aA^
F + P				46.22± 1.34 ^aA^	10.87± 0.45 ^bA^	16.86± 0.27 ^aA^	7.66± 1.70 ^bAB^	46.95± 1.44 ^aA^	9.91 ± 0.15 ^cA^	16.46± 0.63 ^aA^	10.56± 1.73 ^aAB^	47.52± 0.75 ^aA^	10.79± 0.21 ^cA^	16.11± 1.21 ^aA^	10.9± 0.81 ^aAB^	46.66± 0.28 ^bA^	9.55 ± 0.83 ^bB^	15.53± 0.28 ^aB^	11.07± 0.67 ^aA^	46.26± 0.82 ^bA^	9.77 ± 0.48 ^bA^	15.77± 0.43 ^aB^	9.52 ± 0.75 ^bB^

C—unfrozen, unpressurized; F—frozen, unpressurized; P—unfrozen before HPP, pressurized; F + P—frozen before HPP, pressurized. Results and standard errors are mean values of three replicates. Different letters (a, b, c, …) in the same column indicate differences among values. Different letters (A, B, C, …) in the same row indicate differences among values (*p* < 0.05).

**Table 3:** Textural properties of fresh minced meat that was pressurized (350 MPa, 10 °C, 10 min) during storage.

		Day 1	Day 3	Day 7	Day 15	Day 30
Hardness (g)	C	1646.8 ± 108.1 ^bA^	1122.5 ± 124.4 ^bB^	1198.1 ± 29.9 ^bB^	1129.6 ± 71.2 ^cB^	1076.2 ± 48.9 ^bB^
	F	1181.6 ± 72.82 ^cA^	1050.3 ± 150.8 ^bA^	967.4 ± 109.4 ^bA^	1155.2 ± 169.2 ^cA^	983.3 ± 293.6 ^bA^
	P	2087.0 ± 95.2 ^bA^	2061.3 ± 96.6 ^aA^	2280.8 ± 29.9 ^aA^	1633.2 ± 132.1 ^bB^	2148.2 ± 130.3 ^aA^
	F + P	2724.3 ± 402.4 ^aA^	2414.7 ± 311.4 ^aA^	2625.7 ± 109.4 ^aA^	2225.5 ± 172.0 ^aA^	1992.3 ± 17.5 ^aB^
Gumminess (g)	C	1084.5 ± 53.8 ^bA^	720.1 ± 68.6 ^bB^	814.2 ± 8.2 ^bB^	1183.8 ± 81.9 ^bA^	1108.5 ± 12.3 ^bA^
	F	729.1 ± 244.3 ^bA^	934.2 ± 85.1 ^bA^	660.8 ± 37.9 ^bA^	819.6 ± 43.5 ^cA^	832.1 ± 52.8 ^cA^
	P	1460.8 ± 95.40 ^aB^	1477.8 ± 81.8 ^aB^	1712.8 ± 104.1 ^aA^	1251.0 ± 228.7 ^bB^	1549.4 ± 184.2 ^aB^
	F + P	1959.5 ± 96.2 ^aA^	1794.6 ± 180.4 ^aA^	1641.2 ± 121.0 ^aA^	1623.9 ± 70.0 ^aB^	1460.0 ± 101.6 ^aB^
Chewability (g)	C	904.5 ± 47.6 ^cA^	510,4 ± 30,5 dD	610.6 ± 44.41 ^cC^	765.04 ± 62.1 ^cB^	746.2 ± 45.5 ^bB^
	F	679.5 ± 46.3 d^AB^	850,9 ± 98,1 cA	554.2 ± 44.68 ^cB^	626.82 ± 82.6 ^cB^	640.0 ± 101.9 ^bB^
	P	1360.8 ± 105.1 ^bA^	1272.4 ± 64.4 ^bA^	1248.5 ± 120.8 ^bA^	1059.10 ± 78.8 ^bB^	1065.5 ± 98.0 ^aB^
	F + P	1907.6 ± 54.9 ^aA^	1634.3 ± 44.5 ^aB^	1514.8 ± 50.10 ^aB^	1372.7 ± 96.9 ^aAB^	1357.0 ± 96.6 ^aB^
Adhesiveness (g*s)	C	−144.2 ± 4.8 ^bA^	−81.3 ± 9.5 ^cC^	−105.3 ± 8.7 ^bB^	−154.1 ± 8.1 ^bA^	−161.2 ± 9.50 ^bA^
	F	−66.9 ± 4.5 ^cB^	−143.8 ± 41.8 ^bA^	−116.5 ± 10.0 ^bA^	−134.4 ± 10.1 ^bA^	−140.1 ± 5.06 ^bA^
	P	−178.6 ± 12.7 ^aB^	−149.5 ± 6.3 ^bB^	−156.1 ± 22.6 ^aB^	−181.3 ± 19.8 ^aB^	−313.1 ± 21.8 ^aA^
	F + P	−152.7 ± 9.6 ^bB^	−187.9 ± 8.2 ^aA^	−135.7 ± 8.5 ^aB^	−189.9 ± 24.5 ^aA^	−226.0 ± 10.6 ^aA^
Elasticity (mm)	C	0.82 ± 0.04 ^bA^	0.69 ± 0.07 ^bA^	0.74 ± 0.10 ^bA^	0.64 ± 0.11 ^bA^	0.64 ± 0.13 ^bA^
	F	0.82 ± 0.04 ^bA^	0.84 ± 0.05 ^aA^	0.77 ± 0.09 ^bA^	0.77 ± 0.12 ^aA^	0.75 ± 0.10 ^bA^
	P	0.92 ± 0.07 ^aA^	0.86 ± 0.03 ^aA^	0.82 ± 0.03 ^aA^	0.80 ± 0.05 ^aA^	0.93 ± 0.06 ^aA^
	F + P	0.95 ± 0.02 ^aA^	0.94 ± 0.04 ^aA^	0.94 ± 0.03 ^aA^	0.83 ± 0.05 ^aB^	0.91 ± 0.05 ^aA^
Adherence	C	0.66 ± 0.02 ^aA^	0.65 ± 0.02 ^aA^	0.66 ± 0.05 ^aA^	0.64 ± 0.04 ^bA^	0.62 ± 0.04 ^bA^
	F	0.64 ± 0.06 ^aA^	0.64 ± 0.02 ^aA^	0.69 ± 0.04 ^aA^	0.70 ± 0.04 aA	0.71 ± 0.06 ^aA^
	P	0.69 ± 0.06 ^aA^	0.70 ± 0.04 ^aA^	0.71 ± 0.06 ^aA^	0.78 ± 0.06 ^aA^	0.71 ± 0.04 ^aA^
	F + P	0.72 ± 0.05 ^aA^	0.71 ± 0.06 ^aA^	0.73 ± 0.07 ^aA^	0.71 ± 0.02 ^aA^	0.73 ± 0.02 ^aA^
Flexibility (%)	C	0.23 ± 0.03 ^bA^	0.21 ± 0.03 ^bA^	0.25 ± 0.02 ^bA^	0.26 ± 0.01 ^bA^	0.26 ± 0.02 ^bA^
	F	0.22 ± 0.01 ^bA^	0.22 ± 0.01 ^bA^	0.24 ± 0.01 ^bA^	0.23 ± 0.02 ^bA^	0.22 ± 0.02 ^bA^
	P	0.33 ± 0.01 ^aAB^	0.33 ± 0.02 ^aAB^	0.37 ± 0.02 ^aA^	0.36 ± 0.03 ^aA^	0.29 ± 0.02 ^aB^
	F + P	0.34 ± 0.05 ^aA^	0.32 ± 0.02 ^aA^	0.34 ± 0.04 ^aA^	0.32 ± 0.03 ^aA^	0.28 ± 0.01 ^aA^

C—unfrozen, unpressurized; F—frozen, unpressurized; P—unfrozen before HPP, pressurized; F + P—frozen before HPP, pressurized. Results and standard errors are mean values of three replicates. Different letters (a, b, c, …) in the same column indicate differences among values. Different letters (A, B, C, …) in the same row indicate differences among values (*p* < 0.05).

## Data Availability

Not applicable.
